# Usage and Perceived Side Effects of Personal Protective Measures against Mosquitoes among Current Users in Delhi

**DOI:** 10.1155/2014/628090

**Published:** 2014-01-28

**Authors:** Charu Kohli, Rajesh Kumar, G. S. Meena, M. M. Singh, Jyotiranjan Sahoo, G. K. Ingle

**Affiliations:** Department of Community Medicine, Maulana Azad Medical College, New Delhi 110002, India

## Abstract

*Background*. Mosquito-borne diseases constitute an important cause of morbidity and mortality. The use of personal protective measures (PPM) like mats, bednets, screening, repellents, liquid vaporizers, mosquito coils, and so forth has been advocated as an effective tool in control of mosquito-borne diseases, but data about the safety profile of personal protective measures is still scarce. *Objective*. To study the usage and side effects of personal protective measures against mosquitoes among current users in Delhi. *Materials and Methods*. A community-based cross-sectional study among 350 adult individuals selected by systematic sampling method. Data was collected using pretested semistructured questionnaire after taking written informed consent. Data was analysed using SPSS version 17. Chi-square/Fisher's Exact test was used for qualitative variables to find association and *P* value <0.05 was considered significant. *Results*. Out of 350 families selected, 210 belonged to rural area and 140 to urban area. Personal protective measures were used by 219 (62.5%) subjects. Liquid vaporizer was the most preferred method (41.4%). Most common perceived side effect of personal protective measures was headache (7.7%). Other perceived side effects were cough (3.2%), sore throat (2.7%), allergy (1.3%), and eye irritation (0.9%) predominantly among coil users. *Conclusion*. There is a need to have a close watch for side effects of personal protective measures among users. Further research is also needed to develop safe and effective personal protective measures against mosquitoes.

## 1. Introduction

The vector-borne diseases especially those spread through mosquitoes constitute an important cause of morbidity and mortality. In recent years, mosquito-borne diseases have emerged as a serious public health problem in countries of South East Asia Region (SEAR) including India. Nearly half of the world's population is at risk of malaria. It is a leading cause of death especially among Sub-Saharan Africa [1]. Indian scenario is bad as more than three-fourth of population lives in malaria risk areas with 1.86 million disability adjusted life years (DALYs) lost annually [2]. Similarly, dengue which is endemic in around 112 countries worldwide is on a rising trend affecting mainly urban areas of tropical and subtropical regions with about 2.5 billion people at risk of acquiring infection and Indian metropolitan cities and towns are no exception [2–4]. To make the situation even worse in India, chikungunya, Japanese encephalitis, and filariasis outbreaks occur from time to time almost throughout the country. To achieve effective control of mosquito-borne diseases, general public health measures have to be instituted. The use of personal protective measures (PPM) like mats, bed nets, screening, repellents, liquid vaporizers, mosquito coils, and so forth has been advocated as an effective tool in control of mosquito-borne diseases [2]. But data about the safety profile of personal protective measures is still scarce. Animal and some human studies have shown ill effects of personal protective measures [5–7]. Keeping the above aspects in view, this study was planned with an objective to study the usage and perceived side effects of personal protective measures used against mosquitoes among current users in Delhi.

## 2. Materials and Methods

This was a community-based cross-sectional study conducted in a rural area, Barwala located in northwest district, and an urban slum, Balmiki Basti situated in central district in Delhi. The sampling universe included all adult individuals (more than or equal to 18 years) residing in the above-mentioned areas. Sample size was calculated on the basis of results of a previous study where usage of personal protective measures was 54%. Taking 10% allowable error, sample size came out to be 328 [8]. The study sample included 210 individuals from rural area and 140 from urban area (total of 350 adult individuals) selected as per proportion to population size. Systematic random sampling method was used to select study subjects in the study areas. The study was carried out over a period of 1 year from January to December 2012. Data was collected by interviewing the study subjects, after getting written informed consent, using a pretested semistructured interview schedule in the local language. The schedule contained items on socioeconomic and demographic profile of study subjects, usage and perceived side effects of personal protective measures against mosquitoes among users. Current users were defined as those using PPM for at least last one year.

Study was conducted by house-to-house survey. From each family, one individual was selected. Every effort was made to interview the head of the family. In case head of the family was not available, then any person more than 18 years of age was selected using lottery method. When house was found locked even after three visits, next house was selected without disturbing the overall sampling procedure.

Data was analysed using SPSS software (version 17). Results were presented in proportion and mean ± SD wherever applicable. Chi-square test/Fisher's Exact test was used for finding an association between qualitative variables and *P* value less than 0.05 was considered significant. Ethical clearance was taken from institutional ethical committee.

## 3. Results

As shown in Table 1, 147 (42%) subjects were males and 203 (58%) were females. 108 (51.4%) subjects in rural and 55 (39.3%) in urban area belonged to 18–29 years of age group; 59 (28.1%) in rural and 41 (29.3%) in urban area belonged to 30–39 years of age group. 51 (24.3%) subjects in rural area and 33 (23.6%) in urban area were illiterate. Those educated up to middle school were 46 (21.9%) in rural and 30 (21.4%) in urban area. In rural area, 50 (23.8%) subjects were unskilled workers but, in urban area, 39 (27.9%) of subjects were semiskilled workers. Monthly per capita income (in Indian Rupees) in rural area was Rs. 3824.84 ± 2308.41 (SD) and 2846.92 ± 1708.12 (SD) in urban area which ranged from Rs. 1000 to 15,000 in rural and Rs. 666 to 10,000 in urban area.

Study subjects reported use of more than one method for personal protection in their families. 87 (41.4%) families in rural area and 63 (45.0%) in urban areas were using one PPM against mosquitoes. 31 (14.8%) in rural and 18 (12.8%) in urban area reported use of 2 different personal protective methods. 14 (6.7%) in rural and 6 (4.3%) in urban area were using 3 or more PPM. 78 (37.1%) subjects in rural and 53 (37.8%) subjects in urban area reported that they were not using any PPM.

Table 2 shows results of use of personal protective measures against mosquitoes among study subjects. In both rural and urban areas, liquid vaporizers were the most preferred method (41.4%), 46.7% subjects in rural and 33.6% in urban area (*P* = 0.01). The second most common method was coils, used by 44 (21.0%) in rural and 42 (30.0%) in urban area (*P* = 0.05).

Table 3 shows perceived side effects of different personal protective measures. The most common side effect was headache reported by 17 (7.7%) subjects. Other perceived side effects were cough (3.2%), sore throat (2.7%), allergy (1.3%), and eye irritation (0.9%) predominantly among coil users. Among those using coils, 11 (12.8%) reported headache, 7 (8.1%) cough, 5 (5.8%) sore throat, 2 (2.3%) allergy, 2 (2.3%) eye irritation and 1 (1.2%) rashes. Liquid vaporizers were associated with headache 5 (3.4%) and sore throat 1 (0.7%). No side effects were reported with bed nets, repellent creams, and smoke.

## 4. Discussion

In the present study, the most preferred method in both rural (46.7%) and urban areas (33.6%) was liquid vaporizer. A total of 62.5% subjects were using any one of the PPM. These figures are low as compared to a study done in Kerala which found that most (80.0%) of the rural and all of the urban households reported using at least one personal protective measure against mosquitoes. In that study, area was a predictor variable for use of personal protective measures as found in the present study [9]. Present study did not find much use of smoke and other traditional methods like insect repellent plants even in rural area. They have been replaced by modern chemical methods for protection against mosquitoes probably due to their convenience. Ample knowledge and usage concerning traditional insect/mosquito repellent plants are other factors of interest. Application of smoke (91.5%) was one of the most commonly known methods amongst local community by burning the plant parts such as leaves, stems, and roots as discussed by Karunamoorthi et al. [10].

The most common perceived side effect was headache which was felt by 7.7% of the study subjects. Cough (3.2%), sore throat (2.7%), allergy (1.3%), and eye irritation (0.9%) were other side effects reported. In another study carried out by Sharma VP, it was found that 11.8% of people using various types of repellents complained of ill health effects. Breathing problems were the most common (4.2%), followed by eye irritation (2.8%), and often accompanied by bronchial irritation, headache, or skin reaction. Cough, cold, and running nose were accompanied with fever or sneezing in 1.6% cases [11]. Similar findings were reported by another study conducted in Pondicherry where perceived side effects of Personal protective measure were allergy, breathing problems, cough, and headache [12]. Most of the side effects were associated with use of coils. Liquid vaporizers were also associated with headache and sore throat. No side effects were found with repellent creams, bed nets, and smoke.

## 5. Conclusion

There is a need to have a close watch for side effects of personal protective measures among users. Further research is also needed to develop safe and effective personal protective measures against mosquitoes.

## Figures and Tables

**Table 1:** Demographic characteristics of study population.

Characteristics	Rural no. (%) (*N* = 210)	Urban no. (%) (*N* = 140)	Total no. (%) (*N* = 350)
Gender			
Male	86 (41.0)	61 (43.6)	147 (42.0)
Female	124 (59.0)	79 (56.4)	203 (58.0)
Age group (in years)			
18–29	108 (51.4)	55 (39.3)	163 (46.6)
30–39	59 (28.1)	41 (29.3)	100 (28.6)
40–49	28 (13.3)	23 (16.4)	51 (14.6)
50–59	9 (4.3)	12 (8.6)	21 (6.0)
60 and above	06 (2.9)	09 (6.4)	15 (4.3)
Education			
Illiterate	51 (24.3)	33 (23.6)	84 (24.0)
Primary	28 (13.3)	27 (19.3)	55 (15.7)
Middle	46 (21.9)	30 (21.4)	76 (21.7)
High school	42 (20.0)	30 (21.4)	72 (20.6)
Posthigh school and above	43 (20.5)	20 (14.3)	63 (18.0)
Occupation			
Unemployed	17 (8.1)	14 (10.0)	31 (8.9)
Unskilled worker	50 (23.8)	29 (20.7)	79 (22.6)
Semiskilled worker	35 (16.7)	39 (27.9)	74 (21.1)
Skilled worker	16 (7.6)	15 (10.7)	31 (8.9)
Semiprofessional	29 (13.8)	16 (11.4)	45 (12.9)
Professional	39 (18.6)	18 (12.9)	57 (16.3)
Others	24 (11.4)	09 (6.4)	33 (9.4)
Monthly per capita income (in INR)	Rs. 3824.84 ± 2308.41 (SD)	2846.92 ± 1708.12 (SD)	3433.67 + 2140.88 (SD)

**Table 2:** Areawise distribution of practices about personal protective measures.

Personal protective measures used*	Rural no. (%) (*N* = 210)	Urban no. (%) (*N* = 140)	Total no. (%) (*N* = 350)	*P* value
Liquid vaporizers	98 (46.7)	47 (33.6)	145 (41.4)	0.01
Coils	44 (21.0)	42 (30.0)	86 (24.6)	0.05
Bednets	15 (7.1)	05 (3.6)	20 (5.7)	0.15
Insecticide spray	7 (3.3)	10 (7.1)	17 (4.9)	0.10
Mats	9 (4.3)	5 (3.6)	14 (4.0)	0.73
Repellent creams**	3 (1.4)	1 (0.7)	4 (1.1)	0.53
Smoke**	1 (0.5)	1 (0.7)	2 (0.6)	1.00
Not using anything	78 (37.1)	53 (37.8)	131 (37.5)	0.19

*Figures are not mutually exclusive.

**Fisher's Exact test was used.

**Table 3:** Perceived side effects with respect to personal protective measures against mosquitoes among current users.

Side effects*	Coils no. (%) *N* = 86	Liquid vaporizers no. (%) *N* = 145	Insecticide spray no. (%) *N* = 17	Mats no. (%) *N* = 14	Total no. (%) *N* = 219
Headache	11 (12.8)	5 (3.4)	0 (0.0)	01 (7.1)	17 (7.7)
Cough	07 (8.1)	0 (0.0)	0 (0.0)	0 (0.0)	7 (3.2)
Sore throat	05 (5.8)	1 (0.7)	0 (0.0)	0 (0.0)	6 (2.7)
Allergy	02 (2.3)	0 (0.0)	1 (5.9)	0 (0.0)	3 (1.3)
Eye irritation	02 (2.3)	0 (0.0)	0 (0.0)	0 (0.0)	2 (0.9)
Rashes	01 (1.2)	0 (0.0)	0 (0.0)	0 (0.0)	1 (0.4)

*Results are not mutually exclusive.
